# Impact of Dietary Tomato Juice on Changes in Pulmonary Oxidative Stress, Inflammation and Structure Induced by Neonatal Hyperoxia in Mice (*Mus musculus*)

**DOI:** 10.1371/journal.pone.0159633

**Published:** 2016-07-20

**Authors:** Sheena Bouch, Richard Harding, Megan O’Reilly, Lisa G. Wood, Foula Sozo

**Affiliations:** 1 Development and Stem Cells Program, Monash Biomedicine Discovery Institute and Department of Anatomy and Developmental Biology, Monash University, Melbourne, Victoria, Australia; 2 Centre for Asthma and Respiratory Diseases, Hunter Medical Research Institute, The University of Newcastle, Newcastle, New South Wales, Australia; National Institutes of Health, UNITED STATES

## Abstract

Many preterm infants require hyperoxic gas for survival, although it can contribute to lung injury. Experimentally, neonatal hyperoxia leads to persistent alterations in lung structure and increases leukocytes in bronchoalveolar lavage fluid (BALF). These effects of hyperoxia on the lungs are considered to be caused, at least in part, by increased oxidative stress. Our objective was to determine if dietary supplementation with a known source of antioxidants (tomato juice, TJ) could protect the developing lung from injury caused by breathing hyperoxic gas. Neonatal mice (C57BL6/J) breathed either 65% O_2_ (hyperoxia) or room air from birth until postnatal day 7 (P7d); some underwent necropsy at P7d and others were raised in room air until adulthood (P56d). In subsets of both groups, drinking water was replaced with TJ (diluted 50:50 in water) from late gestation to necropsy. At P7d and P56d, we analyzed total antioxidant capacity (TAC), markers of oxidative stress (nitrotyrosine and *heme oxygenase-1* expression), inflammation (*interleukin-1β* (*IL-1β*) and *tumor necrosis factor-α* (*TNF-α*) expression), collagen (*COL*) and smooth muscle in the lungs; we also assessed lung structure. We quantified macrophages in lung tissue (at P7d) and leukocytes in BALF (at P56d). At P7d, TJ increased pulmonary TAC and *COL1α1* expression and attenuated the hyperoxia-induced increase in nitrotyrosine and macrophage influx; however, changes in lung structure were not affected. At P56d, TJ increased TAC, decreased oxidative stress and reversed the hyperoxia-induced increase in bronchiolar smooth muscle. Additionally, TJ alone decreased *IL-1β* expression, but following hyperoxia TJ increased *TNF-α* expression and did not alter the hyperoxia-induced increase in leukocyte number. We conclude that TJ supplementation during and after neonatal exposure to hyperoxia protects the lung from some but not all aspects of hyperoxia-induced injury, but may also have adverse side-effects. The effects of TJ are likely due to elevation of circulating antioxidant concentrations.

## Introduction

Due to improvements in neonatal clinical care, most preterm infants born after 25 weeks of gestation are now capable of survival. However, many preterm infants, especially those born before 32 weeks of gestation, are at high risk of developing bronchopulmonary dysplasia (BPD) [1, 2]. BPD is a form of lung injury involving increased inflammation [3, 4] and is characterized by alterations in the structure and function of both the gas-exchanging tissue of the lung and the conducting airways [5, 6]. It is now recognized that the lung injury and altered lung development associated with BPD increases the risk of persistent respiratory morbidities such as impaired lung function [7, 8] and asthma [9, 10], as well as reduced exercise capacity [11, 12].

In spite of advances in the respiratory management of very preterm infants, the incidence of BPD has not significantly decreased [2]. Approximately 90% of very- and extremely-preterm infants require some form of supplemental oxygen therapy [13] and it is now established that prolonged exposure to supplemental oxygen is a major contributor to BPD [14]. In support of the clinical findings, numerous experimental studies have demonstrated that prolonged inhalation of hyperoxic gas at a time when the lung is still developing can replicate hallmark features of BPD, namely alveolar simplification, increased airway smooth muscle, and inflammation within the lung [15–21].

An important factor contributing to lung injury and altered lung development induced by neonatal hyperoxia is thought to be an increase in oxidative stress

[22]. Preterm infants are likely to be particularly vulnerable to oxidative stress as they have an immature endogenous antioxidant system and are therefore unable to mount an adequate antioxidant response to a hyperoxic environment [23]. Thus, it has been proposed that antioxidant supplementation may protect preterm infants from the injurious effects of hyperoxia by restoring the redox balance [24, 25]. Attempts have been made to improve antioxidant defense in preterm infants by dietary supplementation with known antioxidants [26]. However, clinical trials and laboratory experiments using single antioxidants have shown them to be largely ineffective in ameliorating hyperoxia-induced lung injury [24, 27–29]. Furthermore, an imbalance between different antioxidants can result in tissue damage [30], suggesting that supplementation with single antioxidants could be harmful. As antioxidant defense involves multiple pathways [30], supplementation with any single antioxidant is unlikely to provide adequate protection against injury caused by oxidative stress. Therefore, it has been proposed that a mixture of antioxidants may be more effective in preventing hyperoxia-induced tissue injury [30]. In this regard, tomato juice (TJ) may be a suitable candidate for dietary antioxidant supplementation as it contains a complex array of antioxidants including anthocyanin, β-carotene, vitamins A, C and E and lycopene [31]. These antioxidants have the ability to scavenge reactive oxygen species (ROS) [32]; lycopene in particular is a potent scavenger due to the large number of double bonds within its structure [33]. Dietary supplementation with TJ has been shown to improve lung function and reduce neutrophil influx in asthmatic adults [34]; it has also been shown, in neonatal rats, to prevent nicotine-induced lung injury [35], which is predominantly mediated by an increase in ROS.

We hypothesized that dietary supplementation with a range of antioxidants contained in commercially available TJ would protect the developing lung from the injurious effects of hyperoxia by increasing antioxidant capacity and decreasing oxidative stress; as oxidative stress and inflammation are intimately linked, and neonatal inhalation of hyperoxic gas has been shown to adversely affect pulmonary immune status [15], we proposed that the effect of hyperoxia on immune status would also be ameliorated. Our objective was to determine whether alterations in oxidative stress, inflammation and lung structure induced by prolonged inhalation of hyperoxic gas during lung development could be prevented by dietary supplementation with TJ, a known source of antioxidants that could safely be administered to preterm infants. Owing to differences in lung development between males and females [36], we also interrogated our data for effects of sex.

## Materials and Methods

Our study was approved by the Monash University Animal Ethics Committee (project number: MARP/2011/061) in accordance with the National Health and Medical Research Council of Australia’s Code of Practice for the Care and Use of Animals for Scientific Purposes.

### Treatment groups

Time-mated pregnant C57BL6/J mice (*Mus musculus*) were randomly assigned to four treatment groups: 1. a group that breathed room air from birth (Air); 2. a group that breathed 65% O_2_ for 7 days after birth (Hyperoxia, Hyp); 3. a group that breathed room air from birth and whose diet was supplemented with TJ (Air+TJ); 4. a group that breathed 65% O_2_ for 7 days after birth and whose diet was supplemented with TJ (Hyp+TJ). In the hyperoxia groups, concentrations of O_2_ and CO_2_ were continuously monitored (Servoflex MiniMP 5200, Servomex, TX) within the housing chambers (floor area 501cm^2^; Tecniplast, Italy). Each litter was housed individually. The mice were provided with food and water (or TJ) *ad libitum*. Mice were health checked daily throughout the experimental period. If mice lost weight (~20% reduction in body weight), stopped eating and/or drinking, or displayed reduced physical activity, they were removed from experimental conditions and euthanized via cervical dislocation. No unexpected deaths were encountered. No invasive experimental procedures were performed in this experiment that required the administration of analgesics or anesthetics to ameliorate animal suffering. Within each treatment group, dams and litters were allocated into short-term (7 days) and long-term (56 days) survival studies. Mice are considered to have reached adulthood by 56 days.

#### Short-term survival study

Mice in the Hyp and Hyp+TJ groups were exposed to 65% O_2_ from ~0.5 days prior to birth until postnatal day 7 (P7d; Hyp, n = 37: 18 males (M), 19 females (F); Hyp+TJ, n = 24: 11M, 13F). The Air and Air+TJ groups breathed room air from birth to P7d (Air, n = 32: 17M, 15F; Air+TJ, n = 27: 15M, 12F). Necropsy was performed at P7d following cervical dislocation and the lungs were collected for analysis.

#### Long-term survival study

Mice in the Hyp and Hyp+TJ long-term survival groups were exposed to 65% O_2_ from birth until P7d, after which they were raised in room air until P56d (Hyp, n = 34: 16M, 18F; Hyp+TJ, n = 23: 11M, 12F). The Air and Air+TJ groups breathed room air from birth to P56d (Air, n = 35: 14M, 21F; Air+TJ, n = 27: 15M, 12F). Mice were weaned at P21d. At P56d, necropsy was performed following cervical dislocation for collection of blood, bronchoalveolar lavage fluid (BALF) and the lungs.

#### Tomato juice supplementation

Starting at 15 days after mating (embryonic day 15; E15d), pregnant mice from the TJ groups were provided with commercially available TJ (Heinz Pty Ltd, Australia). According to the manufacturer, the TJ contained the antioxidants lycopene (59mg/L) and vitamin C (300mg/L). The TJ was diluted in water at a ratio of 50:50; the dams and offspring in the TJ groups had *ad libitum* access to the diluted TJ in place of drinking water. The diluted TJ was prepared daily and the volumes consumed were recorded daily; the daily volume of water consumed by the Air and Hyp groups was also recorded. For the short-term survival cohort, drinking water was replaced with the diluted TJ from E15d to necropsy at P7d. For the long-term survival cohort, the diluted TJ was provided from day 15 of pregnancy (E15d) to P56d. Before birth, offspring were presumed to have received antioxidants from the TJ via the placenta and after birth they received antioxidants via maternal milk or from drinking TJ.

### Plasma and tomato juice carotenoid concentrations

At necropsy at P56d, blood was collected by cardiac puncture and plasma stored at -80°C. Plasma and TJ samples were analyzed to determine concentrations of the carotenoids lycopene, α-carotene, β-carotene, β-cryptoxanthin and lutein/zeaxanthin by high performance liquid chromatography (HPLC), which was conducted in a darkened laboratory under red light at The University of Newcastle, Australia. Plasma samples from offspring in each treatment group were pooled to obtain a sufficient volume for analysis; therefore, only one value per group was obtained. Ethanol:ethyl acetate (1:1) containing the internal standard canthaxanthin was added to each sample, and each sample was then vortexed and centrifuged at 3000xg for 10 minutes at 4°C. The supernatant was removed and collected, and the above process repeated three times, the first two with ethanol:ethyl acetate (1:1) and the third with hexane. The supernatants collected from each centrifugation were pooled and ultra-pure water was added to the mixture, which was then vortexed and centrifuged. The supernatant containing the organic solvents was decanted and any remaining solvents evaporated with nitrogen. The sample pellets were reconstituted in dichloromethane:methanol (1:2 v/v) and chromatography was performed on a Hypersil ODS column (100mm x 2.1m x 5μm) with a flow rate of 0.3mL/min. Carotenoids were analyzed using a mobile phase of acetonitrile:dichloromethane:methanol (containing 0.05% ammonium acetate) (85:10:5 v/v) and a diode array detector (450nm) [37].

### Tissue collection and processing at P7d and P56d

At necropsy, lungs of offspring used for histological, immunohistochemical and immunofluorescent analyses were fixed *in situ* via the trachea (at 25cmH_2_O) with 4% paraformaldehyde (PFA) in 0.1M phosphate buffered saline (PBS; pH 7.4). The lungs were then immersion-fixed with 4% PFA in 0.1M PBS (pH 7.4) for 24h, post-fixed in Zamboni’s fixative (10% v/v 37% formaldehyde, 0.1M phosphate buffer, 15% v/v saturated picric acid) for another 24h, processed in ethanol and xylene and then embedded in paraffin wax. At P7d, the numbers of animals in each group were: Air, n = 24 (13M, 11F); Hyp, n = 29 (14M, 15F); Air+TJ, n = 19 (9M, 10F); Hyp+TJ, n = 16 (8M, 8F). At P56d, animal numbers in each group were: Air, n = 27 (10M, 17F); Hyp, n = 26 (12M, 14F); Air+TJ, n = 19 (9M, 10F); Hyp+TJ, n = 16 (9M, 7F).

Separate animals from each treatment group (n = 6–8 per group) provided samples of lung tissue that were snap-frozen in liquid nitrogen at necropsy and stored at -80°C for subsequent quantitative real-time polymerase chain reaction (qPCR) analyses and determination of total antioxidant capacity (TAC).

### Microscopy analyses

Single sagittal sections (5μm) of both the entire left and right paraffin-embedded lungs were used for histological and immunohistochemical analyses. Sagittal sections (5μm) through the entire right paraffin-embedded lung were used for immunofluorescent analysis. For histological and immunohistochemical analyses, stained sections were examined by light microscopy (Nikon Eclipse E400, Nikon, Japan). Color images were captured at a final magnification of x400 using a digital camera (SPOT Insight 4meg Fire Wire Color Mosaic 14.2 Diagnostic Instruments, MI). Images were analyzed using image analysis software (Image-Pro Plus, version 6.2, Media Cybernetics, MD). For immunofluorescent analysis, stained sections were examined by fluorescent microscopy (Zeiss Imager A1, Zeiss, Germany) and images captured with a digital camera (AxioCam MRc5, Zeiss, Germany) linked to image analysis software (AxioVision, version 4.8, Zeiss, Germany). Images were collected at a final magnification of x200. All analyses were conducted in a blinded manner by the same researcher (S.B).

### Oxidative stress in the lung parenchyma

Nitrotyrosine staining within lung tissue was used as a marker for oxidative stress [38]. Nitrotyrosine is produced in tissue by tyrosine nitration following the formation of peroxynitrite by nitric oxide and the superoxide free radical [39]. Nitrotyrosine was detected using an established immunohistochemical stain [40]. In each of the left and right lungs, three and five fields of view of lung parenchyma (free of large conducting airways and major blood vessels) were analyzed at P7d and P56d, respectively. The area of lung tissue positively stained for nitrotyrosine (identified by brown staining) was determined and expressed as a percentage of the total area of tissue for each field of view.

### Pulmonary *heme oxygenase-1* gene expression

Heme oxygenase-1 (HO-1) is an inducible catabolic enzyme that is upregulated in response to a state of oxidative stress [41]. Using frozen lung tissue collected at P7d and P56d, we performed total RNA extraction followed by cDNA synthesis and qPCR to determine the gene expression of *HO-1* (GenBank accession number NM_010442.2) [40]. At P7d, *HO-1* mRNA levels were normalized against the housekeeping gene *β-actin* (GenBank accession number NM_007393.3) using the comparative delta C_T_ (cycle threshold) method; at P56d, *HO-1* mRNA levels were normalized against the housekeeping gene *18S rRNA* (GenBank accession number NC_000072.6) [40]. Values were expressed as a fold-change relative to the mean mRNA levels of the Air group at the same age (P7d or P56d).

### Total antioxidant capacity of lung tissue

At P7d and P56d, the TAC of small antioxidant molecules including dietary antioxidants and uric acid was measured in samples of frozen lung tissue using a TAC assay kit, according to the manufacturer’s instructions (#ab65329, Abcam, UK). The assay measures the ability of a sample to reduce Cu^2+^ ions to Cu^+^. The Cu^+^ ion is chelated with a colorimetric probe producing an absorbance peak at 560nm. Samples were assayed in conjunction with a series of standards, and TAC determined by interpolation of the standard curve.

### Macrophages in lung tissue at P7d

We assessed macrophages in the lung as they play a key role in inflammation [42]. Macrophages in the lung parenchyma were identified using an immunofluorescent stain for galectin-3 [40]. In the right lung, three fields of view that were free of major airways and blood vessels were analyzed. Fields of view were randomly selected using a blue filter, which does not distinguish positively stained cells. The number of macrophages (identified by red staining) was expressed as a percentage of the total number of cells in the lung parenchyma for each field of view.

### Leukocytes in bronchoalveolar lavage fluid at P56d

We measured the number and type of leukocytes in BALF as they are an indicator of pulmonary innate immunity. BALF was collected at necropsy via cannulation of the trachea and lavaging the airway lumen with 2 x 1mL of saline. Leukocytes within the BALF were isolated, prepared and stained with either 0.4% trypan blue solution or May-Grünwald-Geimsa for the determination of the total number of leukocytes per mL of BALF and differential counts of macrophages, neutrophils, lymphocytes and eosinophils, respectively [40].

### Pulmonary *interleukin-1β (IL-1β)* and *tumor necrosis factor-α (TNF-α)* gene expression

We measured *IL-1β* and *TNF-α* gene expression as an indicator of pulmonary inflammation. Using frozen lung tissue collected at P7d and P56d, we performed total RNA extraction followed by cDNA synthesis and qPCR [40] to determine the gene expression of *IL-1β and TNF-α* (GenBank accession numbers NM_008361.3 and NM_013693.2, respectively; Sigma). At P7d, mRNA levels for the genes of interest were normalized against the housekeeping gene *β-actin* (GenBank accession number NM_007393.3) using the comparative delta C_T_ (cycle threshold) method; at P56d, the mRNA levels were normalized against the housekeeping gene *18S rRNA* (GenBank accession number NC_000072.6) [40]. Values were expressed as a fold-change relative to the mean mRNA levels of the Air group at the same age (P7d or P56d).

### Lung morphometry

Lung tissue fraction and mean linear intercept (MLI; an indicator of alveolar size) were measured using sections of lung parenchyma stained with Masson’s Trichrome. We used 30 fields of view per offspring (15 each from the left and right lungs) that were free of large conducting airways and major blood vessels. The tissue fraction and MLI were determined by superimposing test grids over each field of view [43].

We assessed bronchiolar wall structure as bronchioles are an important determinant of lung function [44] by influencing airflow to and from the alveoli. Bronchioles selected for analysis appeared in near-circular cross-section and had an intact wall that lacked cartilage; selected bronchioles had a basement membrane perimeter (P_BM_) ranging from 200–1000μm (approximate diameter: 64–318μm). Bronchiolar wall variables were expressed in relation to the P_BM_ to adjust for differences in bronchiolar size [45]. Wall structure was analyzed for six bronchioles (3 per lung) from each offspring at P7d and ten bronchioles (5 per lung) from each offspring at P56d; the number of bronchioles analyzed was the maximum number that could be consistently obtained at each time-point that met the selection criteria. We used Masson’s Trichrome stained sections to determine the area of the bronchiolar epithelium [46] and the number of bronchiolar-alveolar attachments (N_BA_); both were expressed in relation to the P_BM_ [46]. Bronchiolar-alveolar attachments were measured because they provide mechanical support to bronchioles [47]. Gordon and Sweet’s Reticular Fiber stain was used to determine the area of types I and III collagen fibers (identified by black staining) in the bronchiolar wall, expressed in relation to the P_BM_ [46]. Immunohistochemistry was performed to determine the proportions of proliferating and ciliated cells in the bronchiolar epithelium, expressed in relation to the total number of bronchiolar epithelial cells, using the markers Ki67 and Foxj1, respectively, as previously described [20]. Airway smooth muscle (ASM) in bronchioles was identified immunohistochemically using a primary antibody against α-smooth muscle actin (α-SMA) [40]. The area of α-SMA in the bronchiolar wall (identified by brown staining) was expressed in relation to the P_BM_.

### Pulmonary expression of *collagen* and *α-SMA* genes

Using frozen lung tissue collected at P7d and P56d, we performed total RNA extraction followed by cDNA synthesis and qPCR [40] to determine the expression of the collagen genes *COL1α1*, *COL1α2* and *COL3α1*, and the smooth muscle marker *α-SMA* (GenBank accession numbers NM_007742.4, NM_007743.3, NM_009930.2 and NM_007392.3, respectively; GeneWorks). At P7d and P56d, the mRNA levels for the genes of interest were normalized against the housekeeping genes *β-actin* (GenBank accession number NM_007393.3) and *18S rRNA* (GenBank accession number NC_000072.6), respectively, using the comparative delta C_T_ (cycle threshold) method [40]. Values were expressed as a fold-change relative to the mean mRNA levels of the Air group at the same age (P7d or P56d).

### Statistical analysis of data

Numerical data are expressed as mean ± SEM. Statistical outliers were identified using Grubbs’ outlier test and removed if detected (alpha = 0.05) [48]. The Shapiro-Wilk test was used to determine if data were normally distributed; if they were not, the data were transformed to produce a normally distributed population. Comparisons between groups were made using a two-way ANOVA, with treatment (p^treatment^) and sex (p^sex^) as factors. If the ANOVA showed statistical significance, a least significant difference *post-hoc* test was performed to identify differences between individual treatment groups. Comparison between treatment groups for the volume of TJ or water consumed over time was made using two-way repeated measures ANOVA. TAC and gene expression of *IL-1β*, *TNF-α*, *COL1α1*, *COL1α2*, *COL3α1* and *α-SMA* were analyzed using a one-way ANOVA using treatment as a factor. Statistical significance was accepted at p<0.05.

## Results

### Consumption of tomato juice and water

The volumes of either TJ or water consumed by the dams between birth (P0d) and weaning (P21d) were not significantly different between treatment groups. The mean volume of liquids consumed by all mice (dams and offspring) over this period was 436.7 ± 59.5mL/day/kg body weight. Between weaning and P56d, the volume of either TJ or water consumed by the offspring was not significantly different between groups. The mean volume of liquids consumed in all groups over this period was 261.9 ± 24.0mL/day/kg body weight.

### Plasma and tomato juice carotenoid concentrations

The lycopene concentration in the plasma of the TJ groups was higher than in the non-TJ groups (Air, 0.0μg/L; Hyp, 0.0μg/L; Air+TJ, 4.6μg/L; Hyp+TJ, 6.1μg/L). The concentration of lycopene in undiluted TJ was 40mg/L; therefore the concentration that was consumed was 20mg/L. Based on the mean volume of liquids consumed by all mice, the dose of TJ was 8.7 ± 1.2mg/day/kg body weight prior to weaning and 5.2 ± 0.5mg/day/kg body weight after weaning. Plasma concentrations of other carotenoids assayed (i.e. α-carotene, β-carotene, β-cryptoxanthin and lutein/zeaxanthin) were 0.0μg/L (i.e. undetectable) in all groups.

### Total antioxidant capacity of lung tissue

**Fig 1:**
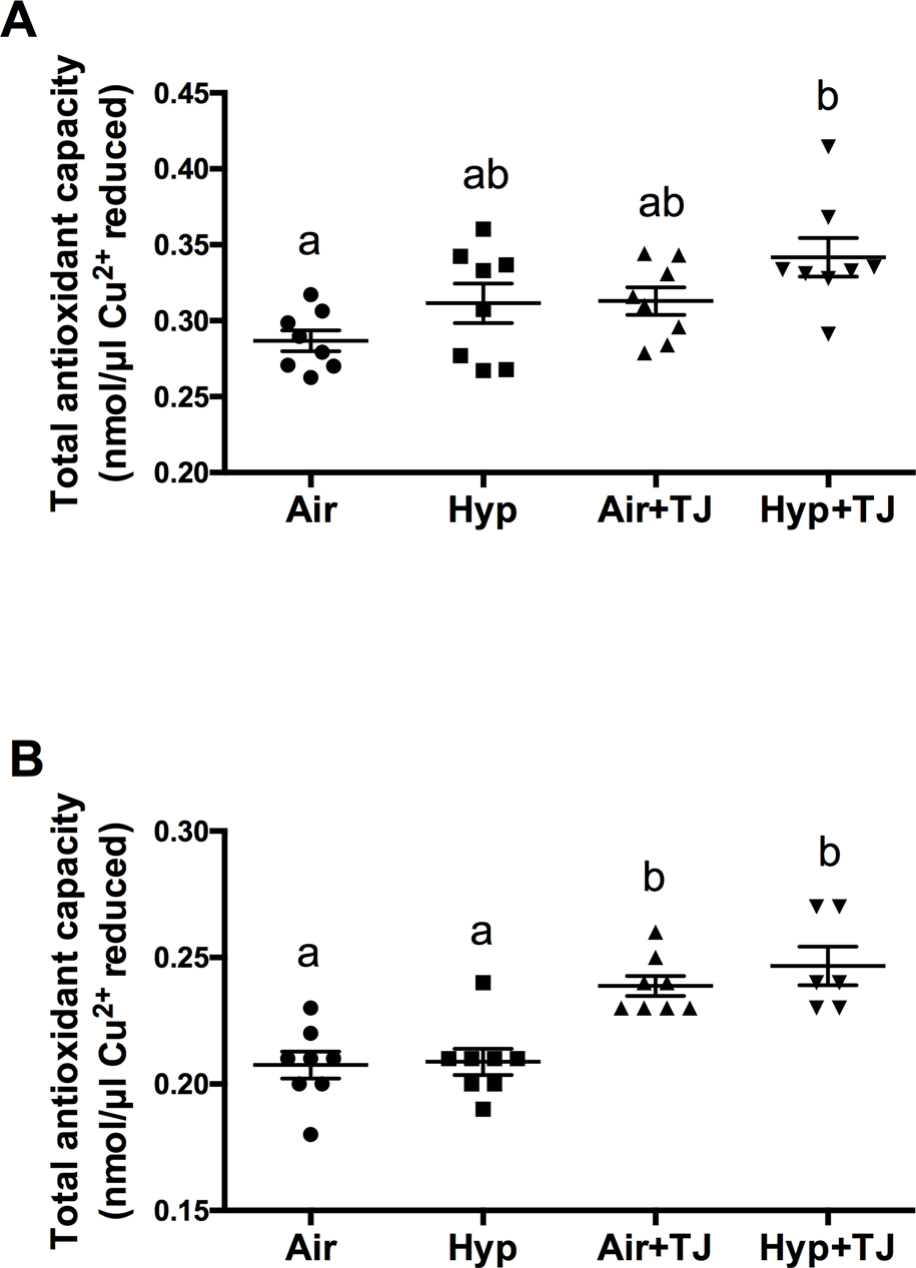
Total antioxidant capacity at P7d and P56d. At P7d, lung TAC was significantly greater in the Hyp+TJ group than in the Air group (**A**). At P56d, lung TAC was significantly greater in both TJ groups than in both non-TJ groups (**B**). Data points represent values from individual animals. Values with different letters are significantly different from each other (p<0.05).

At P7d, the TAC of lung tissue was significantly greater in the Hyp+TJ group than in the Air group (p = 0.001), and there was a trend for it to be greater in the Air+TJ group than in the Air group (p = 0.096). Values (nmol/μl Cu^2+^ reduced) were: Air, 0.29 ± 0.01; Hyp, 0.31 ± 0.01; Air+TJ, 0.31 ± 0.01; Hyp+TJ, 0.34 ± 0.01 (Fig 1A). At P56d, the TAC of lung tissue was significantly greater in both TJ groups than in both non-TJ groups (p<0.001). Values (nmol/μl Cu^2+^ reduced) were: Air, 0.21 ± 0.01; Hyp, 0.21 ± 0.01; Air+TJ, 0.24 ± 0.01; Hyp+TJ, 0.25 ± 0.01, Fig 1B).

### Oxidative stress in the lung

#### *Heme oxygenase-1* gene expression

**Fig 2:**
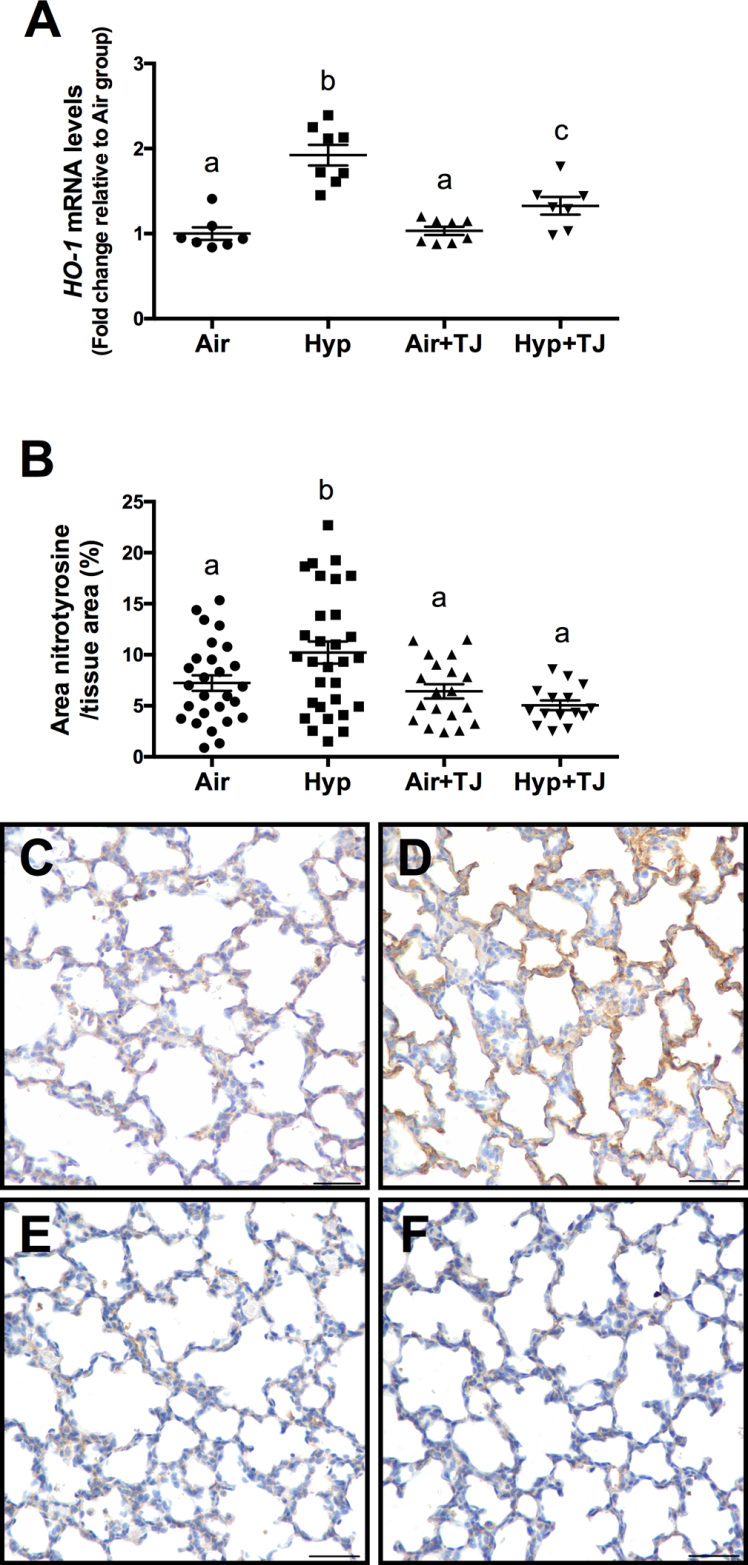
Oxidative stress markers at P7d. The relative gene expression of *heme oxygenase-1* (*HO-1*) in lung tissue was significantly greater in the hyperoxia groups than in the normoxia groups (**A**). The relative gene expression of *HO-1* was significantly lower in the Hyp+TJ group compared to the Hyp group (**A**). The area of nitrotyrosine staining in the lung parenchyma was significantly greater in the Hyp group than in other treatment groups (**B**). Data points represent values from individual animals. Values with different letters are significantly different from each other (p<0.05). Immunohistochemical images (**C**-**F**) of lung sections stained for nitrotyrosine (brown staining) are representative of lung parenchyma analyzed at P7d (**C**, Air; **D**, Hyp; **E**, Air+TJ; **F**, Hyp+TJ). Scale bar = 50μm.

**Fig 3:**
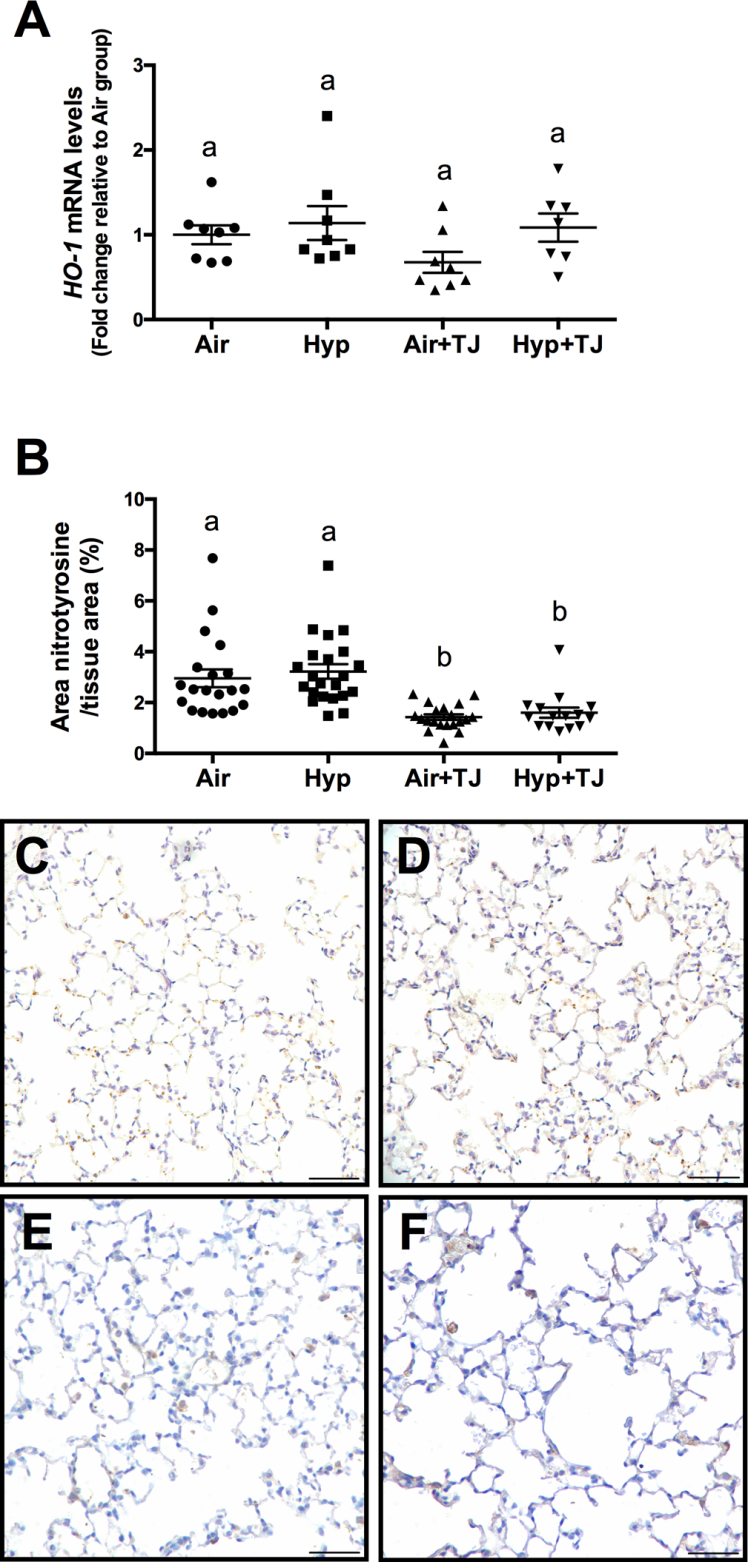
Oxidative stress markers at P56d. The relative gene expression of *heme oxygenase-1* (*HO-1*) was not significantly different between groups (**A**). The area of nitrotyrosine staining in the lung parenchyma was significantly greater in non-TJ groups than in TJ groups (**B**). Data points represent values from individual animals. Values with different letters are significantly different from each other (p<0.05). Immunohistochemical images (**C**-**F**) of lung sections stained for nitrotyrosine (brown staining) are representative of lung parenchyma analyzed at P56d (**C**, Air; **D**, Hyp; **E**, Air+TJ; **F**, Hyp+TJ). Scale bar = 50μm.

At P7d, the relative gene expression of *HO-1* was significantly greater in the hyperoxia groups than in the normoxia groups (p<0.001); however, the relative gene expression of *HO-1* was significantly lower in the Hyp+TJ group compared to the Hyp group (p = 0.000; Air, 1.0 ± 0.1; Hyp, 1.9 ± 0.1; Air+TJ, 1.0 ± 0.1; Hyp+TJ, 1.4 ± 0.1, Fig 2A). At P56d, there was no significant difference between groups in the relative gene expression of *HO-1* (Air, 1.0 ± 0.2; Hyp, 1.1 ± 0.2; Air+TJ, 0.6 ± 0.2; Hyp+TJ, 1.2 ± 0.2, Fig 3A). No sex differences were observed at P7d or P56d.

#### Nitrotyrosine staining in lung tissue

At P7d, the area of nitrotyrosine staining, relative to the total area of lung parenchyma, was significantly greater in the Hyp group compared to the Air, Air+TJ and Hyp+TJ groups (p<0.001; Air, 7.2 ± 0.8%; Hyp, 10.0 ± 0.7%; Air+TJ, 6.7 ± 0.9%; Hyp+TJ, 5.0 ± 1.1%, Fig 2B–2F). At P56d, the relative area of nitrotyrosine staining was significantly greater in the non-TJ groups than in the groups consuming TJ (p<0.001; Air, 2.9 ± 0.3%; Hyp, 3.2 ± 0.3%; Air+TJ, 1.4 ± 0.3%; Hyp+TJ, 1.6 ± 0.3%, Fig 3B–3F). No sex differences were observed at P7d or P56d.

### Leukocytes and inflammatory markers in the lung

#### Galectin-3 staining in lung tissue at P7d

**Fig 4:**
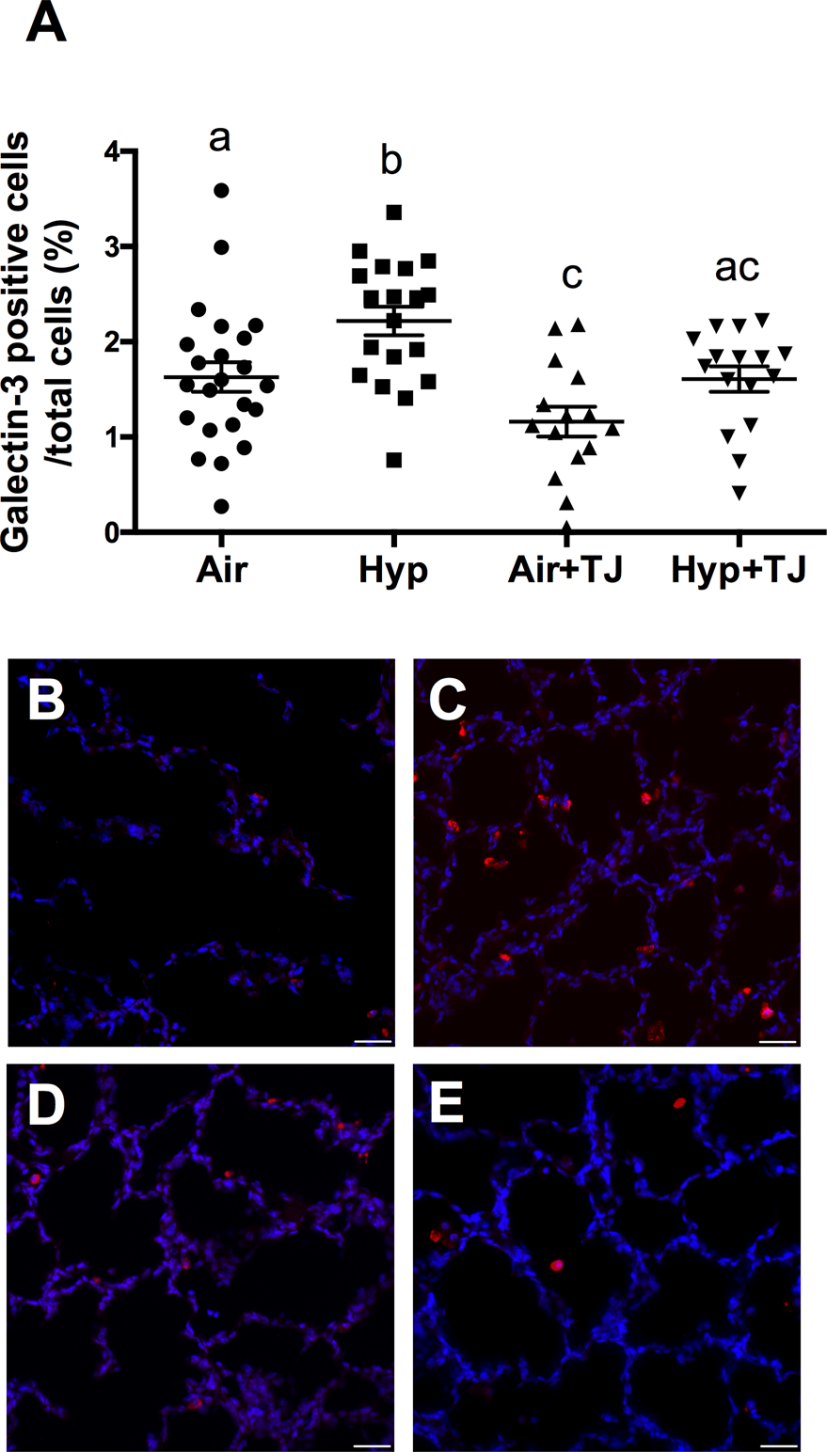
Macrophages in lung tissue at P7d. The proportion of macrophages (galectin-3 positive cells) in the lung parenchyma was significantly greater in the Hyp group than in the Air group; the proportion of macrophages was not significantly different between the two TJ groups (**A**). Data points represent values from individual animals. Values with different letters are significantly different from each other (p<0.05). Immunofluorescent images (**B**-**E**) of lung sections stained for galectin-3 (red staining) are representative of lung parenchyma analyzed at P7d (**B**, Air; **C**, Hyp; **D**, Air+TJ; **E**, Hyp+TJ). Scale bar = 30μm.

The proportion of cells in the lung parenchyma that were macrophages was significantly greater in the Hyp group than in the Air group (p = 0.004); it was significantly lower in the Air+TJ group than in the Air group (p = 0.032), and lower in the Hyp+TJ group compared to the Hyp group (p = 0.007). Values were: Air, 1.7 ± 0.1%; Hyp, 2.2 ± 0.2%; Air+TJ, 1.2 ± 0.2%; Hyp+TJ, 1.6 ± 0.2% (Fig 4A–4E). No sex difference was observed.

#### Leukocytes in bronchoalveolar lavage fluid at P56d

**Fig 5:**
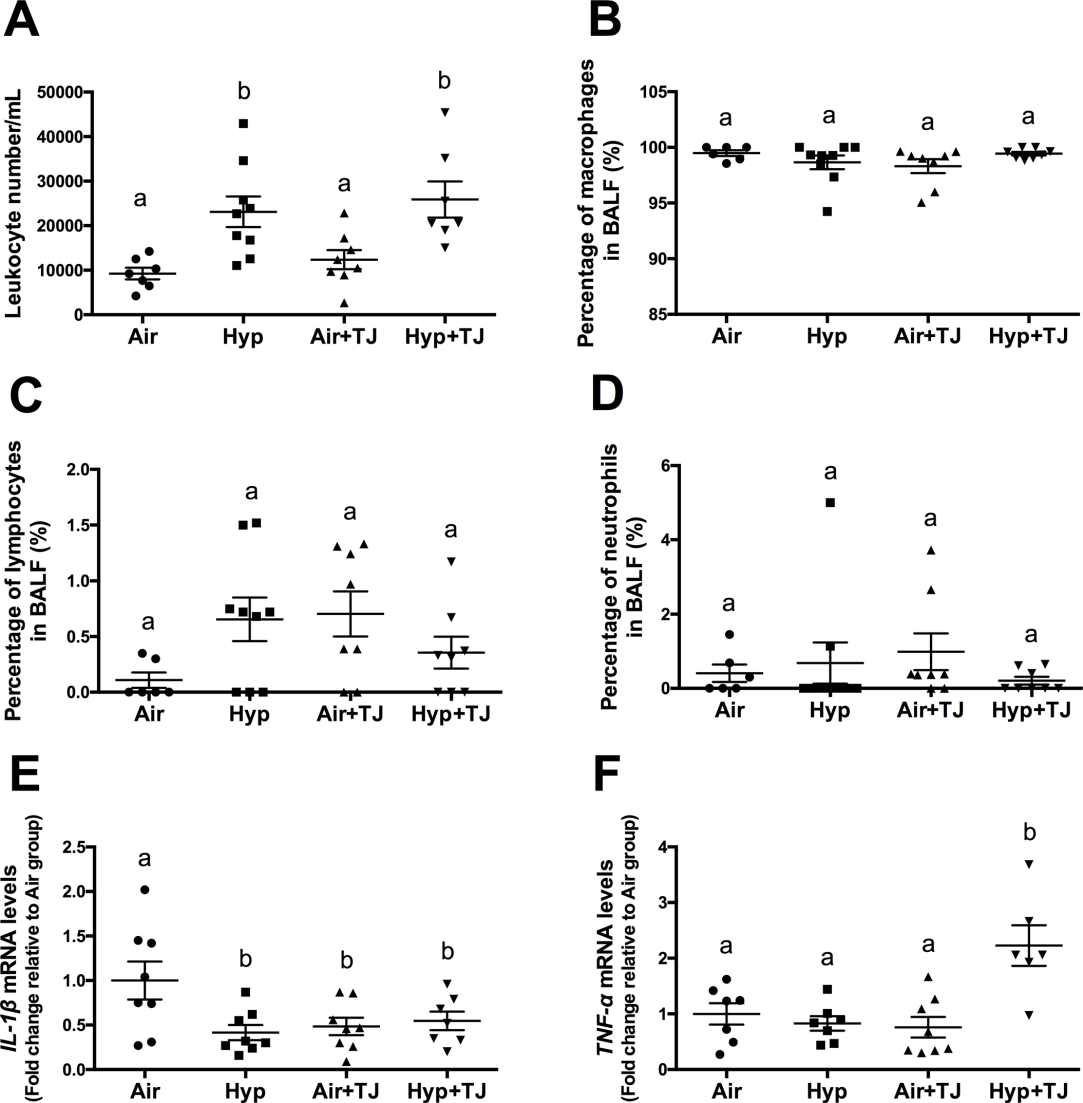
Leukocytes and inflammatory markers in the lung at P56d. The number of leukocytes in bronchoalveolar lavage fluid (BALF) was significantly greater in the hyperoxia groups than in the normoxia groups (**A**). The proportions of macrophages (**B**), lymphocytes (**C**) and neutrophils (**D**) in BALF were not significantly different between the groups. The mRNA expression of *IL-1β* was significantly lower in the Hyp, Air+TJ and Hyp+TJ groups than in the Air group (**E**). The mRNA expression of *TNF-α* was significantly greater in the Hyp+TJ group than in the Air, Hyp and Air+TJ groups (**F**). Data points represent values from individual animals. Values with different letters are significantly different from each other (p<0.05).

The total number of leukocytes per mL of BALF was significantly greater in the hyperoxia groups than in the normoxia groups (p<0.01). There were 150% more leukocytes in the Hyp group compared to the Air group (p = 0.004) and 109% more in the Hyp+TJ group compared to the Air+TJ group (p = 0.006). Values were: Air, 9,238 ± 1,304 cells/mL; Hyp, 23,119 ± 3,453 cells/mL; Air+TJ, 12,371 ± 2,130 cells/mL; Hyp+TJ, 25,878 ± 4,054 cells/mL (Fig 5A). No sex difference was observed.

Enumeration of the different populations of leukocytes in BALF showed that there were no significant differences between treatment groups for the proportion of macrophages (overall mean, 98.93 ± 0.25%), lymphocytes (overall mean, 0.48 ± 0.09%) or neutrophils (overall mean, 0.59 ± 0.21%) (Fig 5B–5D, respectively). No eosinophils were observed in any of the treatment groups. No sex differences were observed.

#### *IL-1β* and *TNF-α gene* expression in lung tissue at P7d and P56d

At P7d, there was a tendency for the mRNA expression of *IL-1β* to be lower in the Air+TJ group than in the other three treatment groups; however, this did not reach statistical significance (p = 0.081; Air, 1.0 ± 0.1; Hyp, 1.1 ± 0.2; Air+TJ, 0.6 ± 0.2; Hyp+TJ, 0.9 ± 0.2, S1A Fig). The mRNA expression of *TNF-α* was not significantly different between the treatment groups (S1B Fig). Values were: Air, 1.0 ± 0.2; Hyp, 1.2 ± 0.2; Air+TJ, 0.8 ± 0.1; Hyp+TJ, 0.8 ± 0.2.

At P56d, the mRNA expression of *IL-1β* was significantly lower in the Hyp, Air+TJ and Hyp+TJ groups compared to the Air group (p = 0.021; Air, 1.0 ± 0.2; Hyp, 0.4 ± 0.1; Air+TJ, 0.5 ± 0.1; Hyp+TJ, 0.5 ± 0.1, Fig 5E). The mRNA expression of *TNF-α* was significantly greater in the Hyp+TJ group compared to the Air, Hyp and Air+TJ groups (p<0.001; Air, 1.0 ± 0.2; Hyp, 0.8 ± 0.1; Air+TJ, 0.8 ± 0.2; Hyp+TJ, 1.6 ± 0.4, Fig 5F).

### Lung morphometry

#### Tissue fraction

At P7d, lung tissue fraction was significantly lower in the hyperoxia groups than in the normoxia groups (p<0.001; Table 1, S2A Fig). At P56d, the tissue fraction was significantly greater in the TJ groups compared to the non-TJ groups (p<0.001; Table 2, S4A Fig). No differences were observed between sexes at P7d or P56d.

**Table 1:** Lung morphometry at P7d.

	**Air**	**Hyp**	**Air+TJ**	**Hyp+TJ**
Tissue fraction (%)*	a: 36.6 ± 1.0	b: 31.9 ± 0.9	a: 39.2 ± 1.2	b: 31.1 ± 1.3
Mean linear intercept (μm)*	a: 113.2 ± 1.9	b: 120.8 ± 1.7	a: 111.2 ± 2.1	b: 125.6 ± 2.3
**Number of bronchiolar-alveolar attachments (N**_**BA**_/mm)*	a: 27.2 ± 0.6	b: 25.6 ± 0.6	a: 27.6 ± 0.7	c: 22.2 ± 0.8
**Epithelial area (μm**^**2**^**/μm)**	a: 6.4 ± 0.2	a: 6.8 ± 0.2	a: 6.3 ± 0.2	a: 6.6 ± 0.2
Epithelial cell proliferation (%)*	a: 18.2 ± 1.5	b: 24.0 ± 1.4	b: 20.8 ± 1.7	b: 21.7 ± 1.9
**Epithelial ciliated cells (%)**	a: 21.3 ± 1.3	a: 18.2 ± 1.2	a: 19.8 ± 1.5	a: 18.7 ± 1.6
**Collagen area (μm**^**2**^**/μm)**	a: 2.8 ± 0.1	a: 2.7 ± 0.1	a: 2.8 ± 0.2	a: 2.7 ± 0.2
*COL1α1* mRNA expression*	a:1.0 ± 0.2	a: 1.0 ± 0.1	b: 3.0 ± 0.5	b: 2.4 ± 0.3
***COL1α2* mRNA expression**	a: 1.0 ± 0.2	a: 0.7 ± 0.2	a: 1.3 ± 0.2	a: 1.5 ± 0.2
***COL3α1* mRNA expression**	a: 1.0 ± 0.2	a: 0.5 ± 0.1	a: 1.2 ± 0.2	a: 1.2 ± 0.2
**α-SMA area (μm**^**2**^**/μm)**	a: 0.9 ± 0.1	a: 1.0 ± 0.1	a: 0.7 ± 0.1	a: 0.7 ± 0.1
***α-SMA* mRNA expression**	a: 1.0 ± 0.1	a: 1.0 ± 0.1	a: 0.6 ± 0.1	a: 0.9 ± 0.2

Morphometric data for lung tissue fraction, mean linear intercept, number of bronchiolar-alveolar attachments, bronchiolar epithelial area, percentage of proliferating and ciliated cells in the bronchiolar epithelium, area of collagen and α-smooth muscle actin (α-SMA) in the outer bronchiolar wall and mRNA expression of *COL1α1*, *COL1α2*, *COL3α1* and *α-SMA* in lung tissue at P7d. Values are mean ± SEM. An asterisk (*) denotes a significant difference between the treatment groups (p<0.05). Values with different letters (before the value) are significantly different from each other.

**Table 2:** Lung morphometry at P56d.

	**Air**	**Hyp**	**Air+TJ**	**Hyp+TJ**
Tissue fraction (%)*	a: 31.5 ± 0.7	a: 30.5 ± 0.7	b: 35.3 ± 0.8	b: 34.1 ± 1.0
**Mean linear intercept (μm)**	a: 86.8 ± 1.3	a: 87.9 ± 1.4	a: 82.6 ± 1.5	a: 84.6 ± 1.8
**Number of bronchiolar-alveolar attachments (N**_**BA**_/mm)*	ac: 28.8 ± 0.6	ab: 29.2 ± 0.6	b: 30.6 ± 0.7	c: 27.3 ± 0.7
**Epithelial area (μm**^**2**^**/μm)**	a: 10.8 ± 0.3	a: 11.5 ± 0.3	a: 11.6 ± 0.3	a: 11.2 ± 0.4
**Epithelial cell proliferation (%)**	a: 2.7 ± 0.3	a: 2.4 ± 0.3	a: 3.4 ± 0.3	a: 2.8 ± 0.4
Epithelial ciliated cells (%)*	a: 27.8 ± 1.1	a: 27.0 ± 1.0	b: 19.9 ± 1.1	b: 20.3 ± 2.2
**Collagen area (μm**^**2**^/μm)*	a: 3.3 ± 0.1	ab: 3.5 ± 0.1	b: 3.8 ± 0.2	b: 3.9 ± 0.2
***COL1α1* mRNA expression**	a: 1.0 ± 0.3	a: 0.4 ± 0.1	a: 0.5 ± 0.1	a: 0.9 ± 0.2
***COL1α2* mRNA expression**	a: 1.0 ± 0.3	a: 0.6 ± 0.1	a: 0.6 ± 0.1	a: 0.8 ± 0.2
***COL3α1* mRNA expression**	a: 1.0 ± 0.2	a: 0.6 ± 0.1	a: 0.7 ± 0.1	a: 1.1 ± 0.2
**α-SMA area (μm**^**2**^/μm)*	a: 0.7 ± 0.1	b: 1.0 ± 0.1	a: 0.6 ± 0.1	a: 0.6 ± 0.1
***α-SMA* mRNA expression**	a: 1.0 ± 0.2	a: 0.7 ± 0.1	a: 0.5 ± 0.1	a: 0.6 ± 0.1

Morphometric data for lung tissue fraction, mean linear intercept, number of bronchiolar-alveolar attachments, bronchiolar epithelial area, percentage of proliferating and ciliated cells in the bronchiolar epithelium, area of collagen and α-smooth muscle actin (α-SMA) in the outer bronchiolar wall and mRNA expression of *COL1α1*, *COL1α2*, *COL3α1* and *α-SMA* in lung tissue at P56d. Values are mean ± SEM. An asterisk (*) denotes a significant difference between the treatment groups (p<0.05). Values with different letters (before the value) are significantly different from each other.

#### Mean linear intercept

At P7d, the MLI was significantly greater in the hyperoxia groups compared to the normoxia groups (p<0.001; Table 1, S2B Fig). At P56d, differences in MLI were no longer apparent (Table 2, S4B Fig). No sex differences were observed at P7d or P56d.

#### Bronchiolar-alveolar attachments

At P7d, the N_BA_, relative to P_BM_, was significantly lower in the hyperoxia groups than in the normoxia groups (p<0.001; Table 1, S2C Fig). The N_BA_ in the Hyp+TJ group was further decreased compared to the Hyp group (p = 0.001; Table 1, S2C Fig). At P56d, N_BA_ was significantly greater in the Air+TJ group compared to the Air group, and lower in the Hyp+TJ group compared to the Air+TJ group (p<0.01; Table 2, S4C Fig). No sex differences were observed at P7d or P56d.

#### Bronchiolar epithelium

At P7d and P56d, the bronchiolar epithelial area was not significantly different between groups (Tables 1 and 2; S2D and S4D Figs, respectively) or sexes. At P7d, the proportion of epithelial cells undergoing proliferation was significantly greater in the Hyp, Air+TJ and Hyp+TJ groups than in the Air group (p<0.01; Table 1, S2E Fig). Sex (p^sex^ = 0.011; males, 19.9 ± 1.4%; females, 22.6 ± 0.9%) and sex-treatment interaction (p^sex*treatment^ = 0.040) effects were observed, with females in the Air (males, 15.4 ± 2.9%; females, 21.0 ± 2.2%) and Hyp+TJ (males, 18.4 ± 2.4%; females, 24.9 ± 1.8%) groups having greater epithelial cell proliferation than males. No significant differences were observed between the sexes in the Hyp and Air+TJ groups. At P56d, the proportion of proliferating epithelial cells did not differ between treatment groups (Table 2, S4E Fig) and no sex difference was observed. At P7d, there were no significant differences between groups in the proportion of ciliated epithelial cells (Table 1, S2F Fig). At P56d, the proportion of ciliated epithelial cells was significantly lower in the TJ groups than in the non-TJ groups (p<0.001; Table 2, S4F Fig). No sex differences were observed at P7d and P56d.

#### Bronchiolar and pulmonary collagen

**Fig 6:**
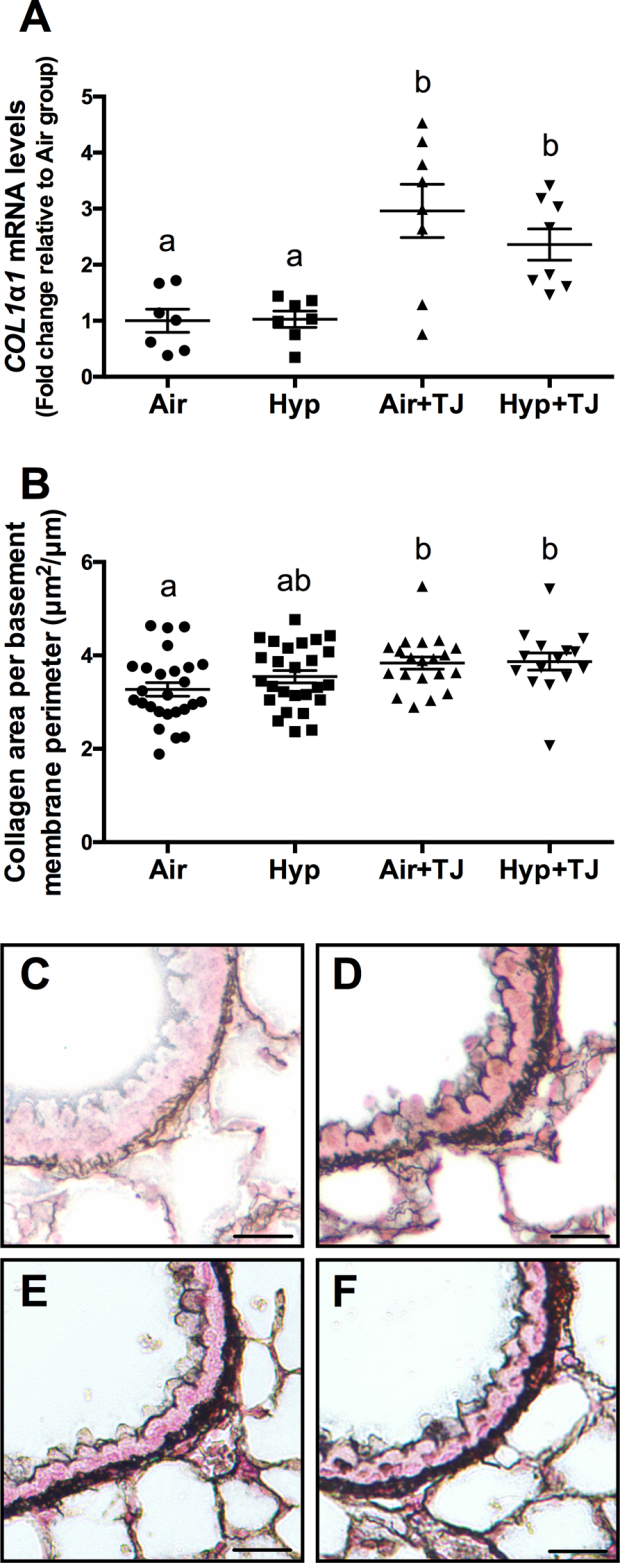
Collagen expression in the lung at P7d and P56d. The mRNA expression of *COL1α1* in lung tissue at P7d was significantly greater in the TJ groups compared to the non-TJ groups (**A**). The area of collagen in the outer bronchiolar wall at P56d was significantly greater in the TJ groups compared to the Air group (**B**). Data points represent values from individual animals and values with different letters are significantly different from each other (p<0.05). Representative images of lung sections stained for collagen (black staining) are representative of the bronchioles analyzed at P56d (**C**, Air; **D**, Hyp; **E**, Air+TJ; **F**, Hyp+TJ). Scale bar = 10μm.

At P7d, the area of collagen in the outer bronchiolar wall did not significantly differ between groups (Table 1, S2G Fig) and no difference was observed between sexes. The gene expression of *COL1α1* in lung tissue at P7d was significantly greater in the TJ groups than in the non-TJ groups (p<0.001; Table 1, Fig 6A). There was a tendency for the gene expression of *COL1α2* (p = 0.062) and *COL3α1* (p = 0.052) to be greater in the TJ groups compared to the Hyp group, but this did not reach statistical significance (Table 1, S3A and S3B Fig). At P56d, the area of collagen in the outer bronchiolar wall in the TJ groups was significantly greater than in the Air group (p<0.05; Table 2, Fig 6B–6F). Collagen area was significantly greater in females than in males (p^sex^ = 0.026; males, 3.5 ± 0.2μm^2^/μm; females, 3.8 ± 0.1μm^2^/μm). The gene expression of *COL1α1*, *COL1α2* and *COL3α1* in lung tissue was not significantly different between groups at P56d (Table 2, S5A–S5C Fig).

#### Bronchiolar and pulmonary α-SMA

**Fig 7:**
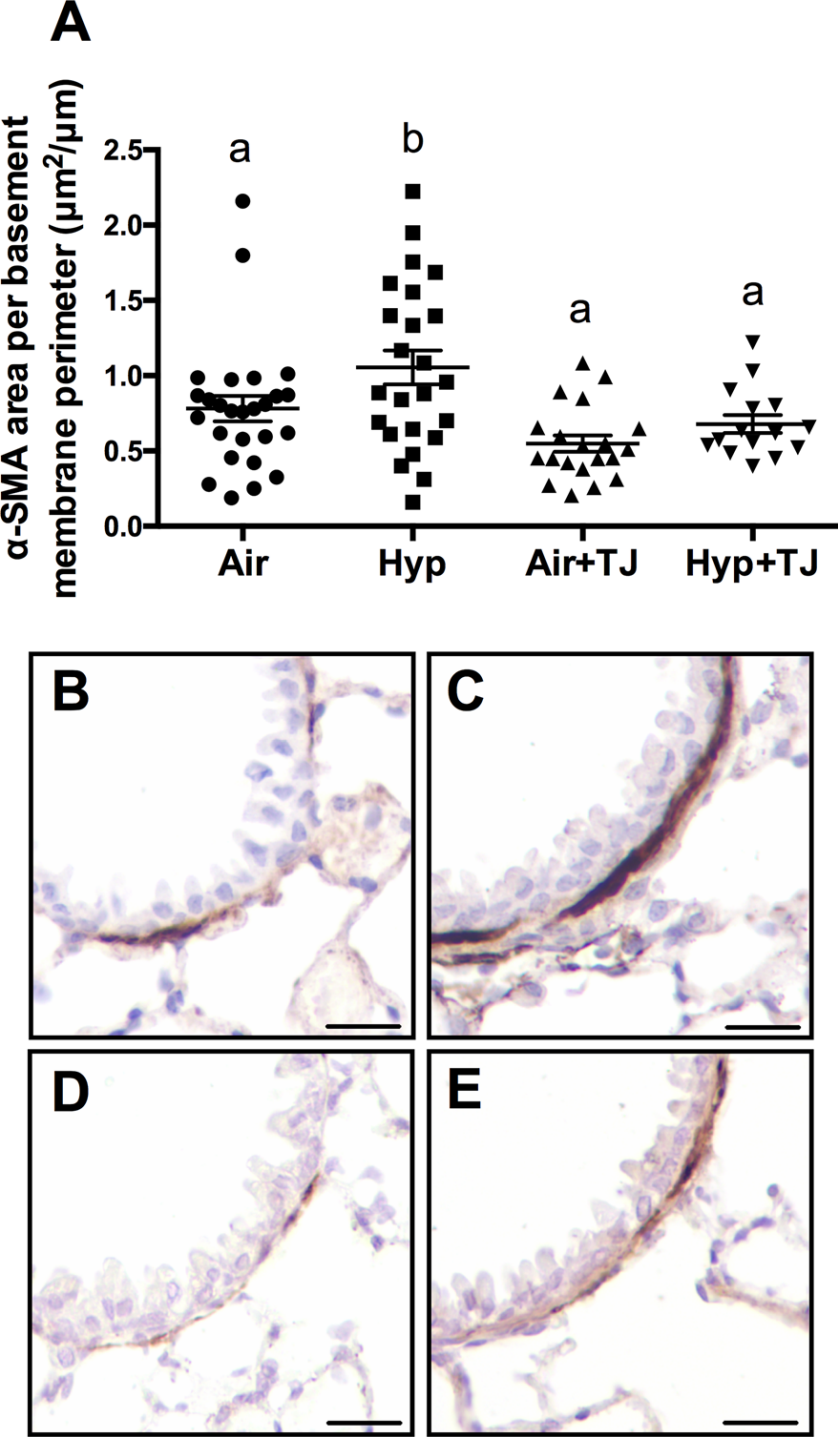
The area of smooth muscle in the outer bronchiolar wall at P56d. The area of smooth muscle was significantly greater in the Hyp group compared to the other treatment groups (**A**). Data points represent values from individual animals and values with different letters are significantly different from each other (p<0.05). Immunohistochemical images (**B**-**E**) of lung sections stained for α-SMA (brown staining) are representative of the bronchioles analyzed at P56d (**B**, Air; **C**, Hyp; **D**, Air+TJ; **E**, Hyp+TJ). Scale bar = 10μm.

At P7d, the area of α-SMA in the outer bronchiolar wall and the gene expression of *α-SMA* in lung tissue did not significantly differ between groups (Table 1, S2H and S3C Figs, respectively). At P56d, the Hyp group had significantly more smooth muscle in the outer bronchiolar wall than the other groups (p<0.001; Table 2, Fig 7). No significant sex differences in the area of α-SMA in the outer bronchiolar wall were observed at P7d or P56d. There was a tendency (p = 0.086) for the gene expression of *α-SMA* to be greater in the Hyp+TJ group than in the Hyp group, but this did not reach statistical significance (Table 2, S5D Fig).

## Discussion

Our study shows that ingestion of TJ attenuates some of the adverse effects of neonatal inhalation of hyperoxic gas. At P7d, TJ ameliorated the hyperoxia-induced increases in the oxidative stress marker nitrotyrosine and in the proportion of macrophages in lung tissue. At P56d, TJ consumption led to increased TAC of the lung tissue and ameliorated the hyperoxia-induced increase in ASM.

### Antioxidant concentrations

Few experimental studies [34, 35, 49, 50] have examined the protective effects of multiple dietary antioxidants on induced alterations in lung development. We used TJ to protect against hyperoxia-induced lung injury, as it is reportedly a readily available source of multiple antioxidants that could potentially be administered to preterm infants [31]. The TJ we used had a measured lycopene concentration of 40mg/L; therefore, the diluted TJ provided to the animals had a lycopene concentration of 20mg/L, which equated to an approximate dose of 7mg/day/kg body weight and led to a plasma lycopene concentration of 5-6ng/L at P56d. The calculated dose of lycopene in our study is equivalent to that used in a previous study that showed a significant protective effect of lycopene supplementation on allergic inflammation in a mouse model of allergic airways disease [51]. There is little evidence regarding the plasma antioxidant concentrations required to exert significant effects *in vivo*. Although other carotenoids were not detected in the plasma of supplemented mice, TJ increased the TAC of small antioxidant molecules in lung tissue; this increase could be due to antioxidants other than lycopene being increased, especially vitamin C as it reportedly has a concentration of 300mg/L in undiluted TJ, which would equate to an approximate dose of 52mg/day/kg body weight. Our TJ dosing regimen was chosen as it could potentially be given to preterm infants and has been shown to protect neonatal rats from nicotine-induced changes in lung architecture, which are largely due to oxidative stress [35]. In future, it may be beneficial to alter the route or timing of TJ administration to further increase the plasma concentrations of antioxidants in TJ, including lycopene and vitamins A, C and E.

### Total antioxidant capacity of lung tissue

We conducted a TAC assay to determine whether TJ led to a functional increase in the TAC of small antioxidant molecules within the lung. At P7d, TAC was significantly greater in the Hyp+TJ group than in the Air group, and there was a tendency for TAC to be increased in the Air+TJ group. These results show that maternal TJ ingestion increases pulmonary TAC in suckling offspring, confirming that the ingested antioxidants are transferred across the placenta and/or are present within the breast milk of the dam. At P56d, the TAC of lung tissue was significantly greater in both of the TJ groups compared to the non-TJ groups. Together, the results obtained at P7d and P56d suggest that dietary TJ increased the lung’s ability to reduce ROS. Furthermore, this increase in TAC was related to evidence of significantly lower oxidative stress in the lungs, as discussed below.

### Oxidative stress in the lung

It is well established that hyperoxia leads to oxidative stress within the lung [49, 52–54], and our observations confirm this effect as there was increased oxidative stress in the Hyp group at P7d. Importantly, *HO-1* was decreased in the Hyp+TJ group compared to the Hyp group, and the hyperoxia-induced increase in nitrotyrosine was inhibited by TJ. Further, a recent study has shown that dietary supplementation with vitamin A and retinoic acid attenuates hyperoxia-induced oxidative stress in the lung [49]. In contrast, the use of single antioxidants is largely ineffective at reducing hyperoxia-induced oxidative stress within the lung [55, 56]. It appears therefore that multiple dietary antioxidants are more effective in reducing ROS, and hence oxidative stress, than single antioxidants.

We found that the antioxidative effects of TJ were present at P56d, with TJ resulting in a decrease in nitrotyrosine deposition in the absence of changes in *HO-1*. *HO-1* and nitrotyrosine are differentially regulated, which is likely the reason for the differing time-course of expression. This appears to be the first demonstration that TJ can induce a decrease in oxidative stress that persists to adulthood. However, this long-term effect may be cause for concern as ROS production is important for normal physiological processes, including cell signaling pathways [57] and host defense [58]. Thus, a depletion of ROS and a reduction in oxidative stress under basal conditions could alter the redox balance within the lung, increasing the redox capacity to an undesirable level, which could be deleterious to normal lung development.

### Leukocytes and inflammatory markers in the lung

At P7d, we found a greater proportion of macrophages in the lung parenchyma in the Hyp group than in the Air group. This is consistent with other studies showing that neonatal hyperoxia leads to an increase in pulmonary leukocytes, including macrophages, immediately after exposure [15, 59]. However, our Hyp+TJ group did not have an increased proportion of macrophages compared to the Air or Air+TJ groups, suggesting that TJ may protect the lung from the hyperoxia-induced influx of macrophages immediately following exposure. Interestingly, in the Air+TJ group, the proportion of macrophages was reduced, suggesting that TJ affects pulmonary immune cell number under normal physiological conditions, which may impair innate immunity. To further characterize the immune response at P7d, we analyzed the gene expression of the pro-inflammatory markers *IL-1β* and *TNF-α* in lung tissue. The finding that there was no significant difference in the expression of *IL-1β* or *TNF-α* between treatment groups was unexpected as macrophages are a major source of *IL-1β* and *TNF-α* in the lung [60, 61] and are an essential component of host defense. However, as we did not interrogate macrophage phenotype and function in the present study, the different proportions of macrophages found between treatment groups could be due to cytokines other than *IL-1β* or *TNF-α* being differentially expressed.

At P56d, we confirmed that neonatal hyperoxia increased the concentration of leukocytes in BALF [40], and that this effect was not ameliorated by TJ. We also found that the gene expression of *IL-1β* was lower in the Hyp, Air+TJ and Hyp+TJ groups than in the Air group and the gene expression of *TNF-α* was greater in the Hyp+TJ group than in the other three treatment groups. As *TNF-α* is known to play a role in M1 differentiation, the macrophage population in the Hyp+TJ group may be skewed towards a M1 phenotype, indicating a potentially detrimental increase in inflammation [62]. Taken together, it appears that TJ did not attenuate the long-term pulmonary inflammatory response to neonatal hyperoxia and may have increased part of the inflammatory response.

### Lung morphometry

Our findings on the effects of hyperoxia on lung tissue fraction, MLI and bronchiolar-alveolar attachments are consistent with previous studies [15, 18, 38, 63]. We confirm that neonatal inhalation of 65% O_2_ inhibits alveolarization, and suggest that TJ does not protect the lung from the adverse effects of hyperoxia on alveolarization. Our results show that the early morphometric effects of hyperoxia (65% O_2_) do not persist to adulthood, apart from a reduction in the number of bronchiolar-alveolar attachments in the Hyp+TJ group. Overall, our findings indicate recovery from the hyperoxia-induced inhibition of alveolarization by adulthood and suggest that TJ may adversely affect the ability of alveolarization to recover from early impairment. As previous studies have observed changes in lung function in adulthood following exposure to neonatal hyperoxia [19], further studies are required to determine whether TJ alters lung function.

Our findings suggest that TJ does not prevent hyperoxia-induced proliferation of bronchiolar epithelial cells at P7d. As the observed increase in bronchiolar epithelial cell proliferation was not accompanied by an increase in epithelial area, it is possible that the rate of epithelial cell apoptosis was increased, leading to increased cell turnover. At P56d, the proportion of ciliated epithelial cells was decreased in both of the TJ groups, suggesting that TJ alone, but not hyperoxia, could be causing this effect. Thus, the TJ supplemented mice may have had a reduced ability to move mucus along their airways.

The gene expression of *COL1α1* in lung tissue and bronchiolar collagen deposition were elevated in the TJ groups relative to the Air group, at P7d and P56d respectively. This TJ-induced increase in collagen was unexpected and could be detrimental to lung function as increased collagen is indicative of pulmonary fibrosis [64] and is observed in asthmatics [65].

An increase in bronchiolar smooth muscle was observed in the Hyp group at P56d, relative to the Air and Hyp+TJ groups. This increased bronchiolar smooth muscle was not accompanied by an increase in the gene expression of *α-SMA* at either P7d or P56d; this finding may have been because α-SMA deposition was only measured in the outer airway wall whereas *α-SMA* gene expression was measured in samples of whole lung tissue. The observed increase in ASM in adult mice following neonatal hyperoxia is consistent with previous studies [17, 19, 66, 67]. Increased ASM is a hallmark feature of BPD [68] and is also implicated in the pathogenesis of obstructive lung diseases, especially asthma [69]. As our data indicate that TJ may prevent the persistent increase in ASM induced by neonatal hyperoxia, it could be beneficial in the management of very preterm infants who are at risk of developing BPD and asthma later in life [1, 70].

### Effects of TJ alone on lung development

Our study revealed differences between the Air and Air+TJ groups at P7d and P56d, suggesting that TJ alone may alter lung development. The potentially adverse effects of TJ observed at P56d may be due to the long duration of TJ ingestion. Our findings suggest that TJ alone can alter cellular and structural development of the lungs, including increasing lung tissue fraction and collagen deposition, and markers of oxidative stress and inflammation under physiological conditions. Therefore, it is plausible that a reduction in ROS resulting from TJ ingestion during normal physiological conditions could adversely affect lung development.

### Conclusions

We conclude that, although TJ does not ameliorate hyperoxia-induced alterations in lung architecture immediately following exposure, it has the beneficial effects of increasing TAC and attenuating the hyperoxia-induced influx of macrophages and increase in oxidative stress in the lungs. Importantly, TJ also protected hyperoxia-exposed lungs from a persistent increase in bronchiolar smooth muscle. However, TJ may alter the persistent pulmonary inflammatory response to neonatal hyperoxia, and it appears that TJ alone adversely affects some aspects of lung cellular and architectural development, redox balance and innate immunity, and may induce fibrosis. In view of the potentially adverse effects of TJ observed in our study, future studies could trial lower dosages, different dosing regimens or other routes of administration.

## Supporting Information

S1 Fig Gene expression of *IL-1β* and *TNF-α* at P7d. mRNA expression of *IL-1β* (**A**) and *TNF-α* (**B**) in lung tissue. Data points represent values from individual animals. Values with the same letter are not significantly different from each other (p<0.05).
(PDF)

S2 Fig Structural analysis of lung morphometry at P7d. Morphometric analyses of tissue fraction (**A**), mean linear intercept (**B**), the number of bronchiolar-alveolar attachments (**C**), the bronchiolar epithelial area (**D**), proportion of proliferating (**E**) and ciliated (**F**) cells in the bronchiolar epithelium, bronchiolar collagen content (**G**), and bronchiolar α-SMA content (**H**). Data points represent values from individual animals. Values with different letters are significantly different from each other (p<0.05).
(PDF)

S3 Fig Gene expression of collagen and smooth muscle markers at P7d. mRNA expression of *COL1α2* (**A**), *COL3α1* (**B**) and *α-SMA* (**C**) in lung tissue. Data points represent values from individual animals. Values with the same letter are not significantly different from each other (p<0.05).
(PDF)

S4 Fig Structural analysis of lung morphometry at P56d. Morphometric analyses of tissue fraction (**A**), mean linear intercept (**B**), the number of bronchiolar-alveolar attachments (**C**), the bronchiolar epithelial area (**D**) and proportion of proliferating (**E**) and ciliated (**F**) cells in the bronchiolar epithelium. Data points represent values from individual animals. Values with different letters are significantly different from each other (p<0.05).
(PDF)

S5 Fig Gene expression of collagen and smooth muscle markers at P56d. mRNA expression of *COL1α1* (**A**), *COL1α2* (**B**), *COL3α1* (**C**) and *α-SMA* (**D**) in lung tissue. Data points represent values from individual animals. Values with the same letter are not significantly different from each other (p<0.05).
(PDF)
