# Supplementary figures and images for: Impact of Dietary Tomato Juice on Changes in Pulmonary Oxidative Stress, Inflammation and Structure Induced by Neonatal Hyperoxia in Mice (Mus musculus)
Source: PLoS One. 2016 Jul 20;11(7):e0159633. doi: 10.1371/journal.pone.0159633 (PMC4954692; doi:10.1371/journal.pone.0159633)

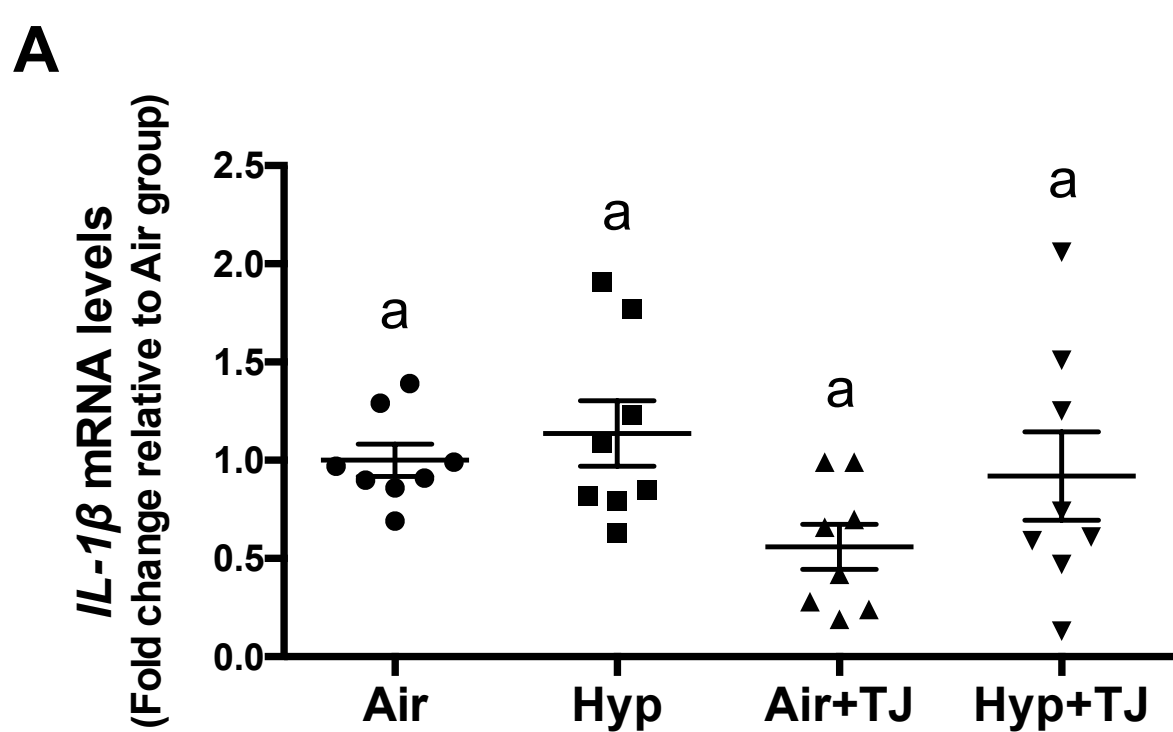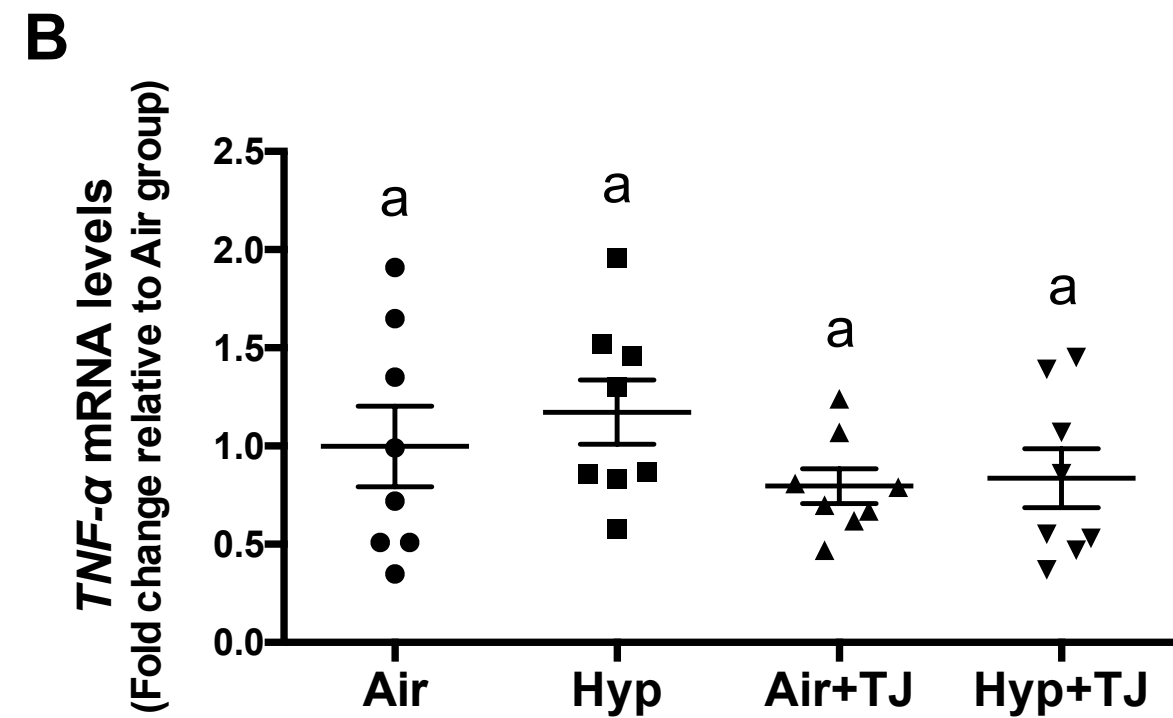

Supplement: S1 Fig — mRNA expression of IL-1β (A) and TNF-α (B) in lung tissue. Data points represent values from individual animals. Values with the same letter are not significantly different from each other (p<0.05). (PDF) [file pone.0159633.s001.pdf]

**A**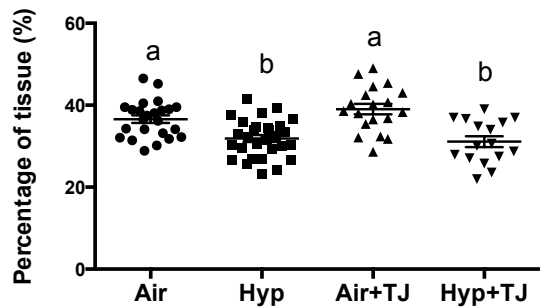**B**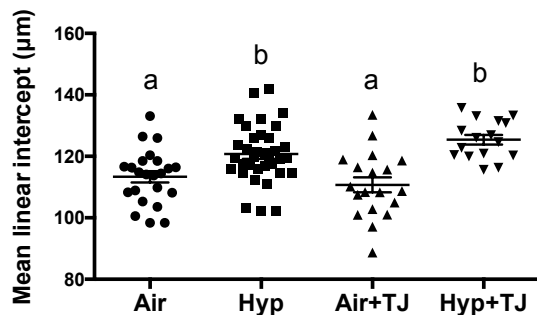**C**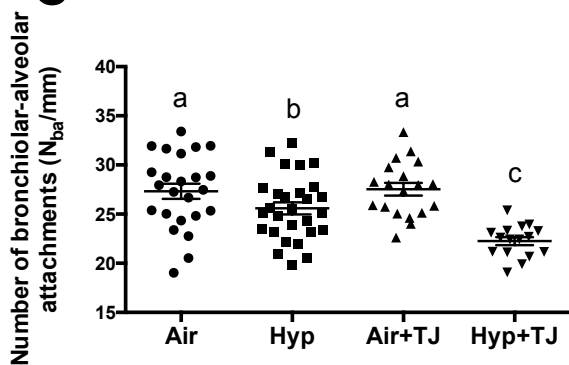**D**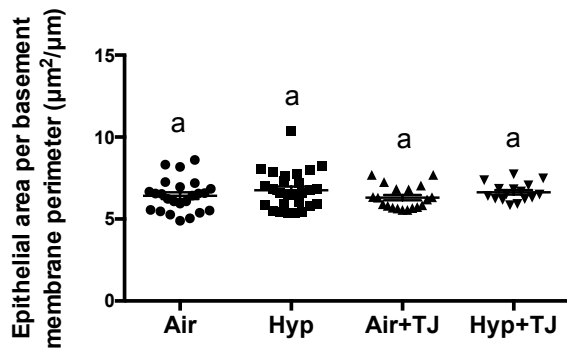**E**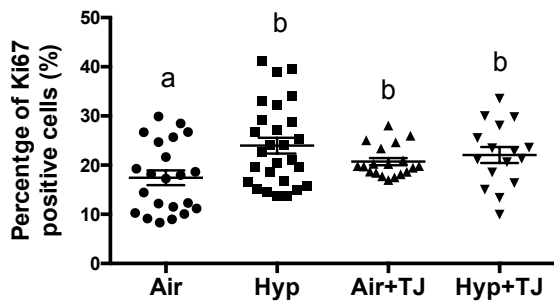**F**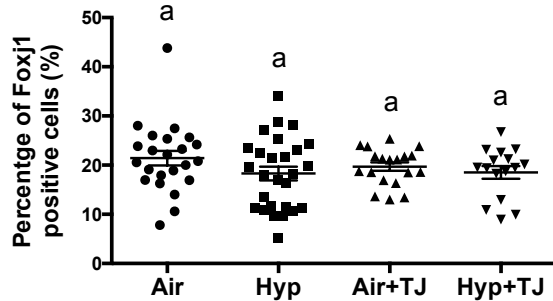**G**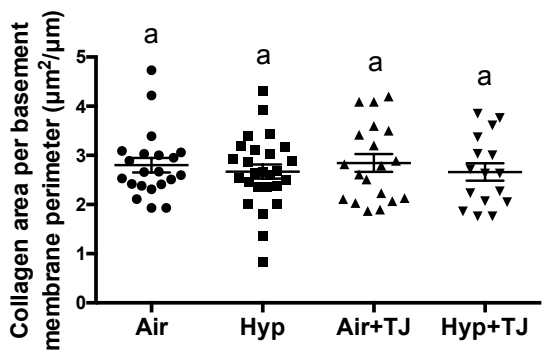**H**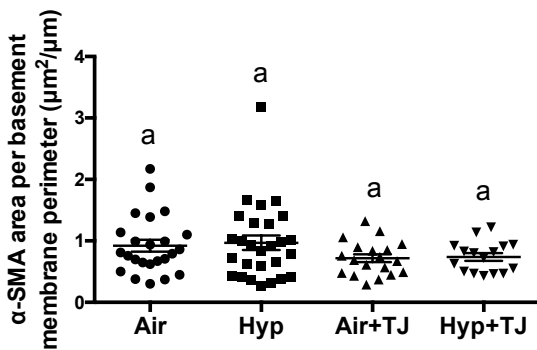

Supplement: S2 Fig — Morphometric analyses of tissue fraction (A), mean linear intercept (B), the number of bronchiolar-alveolar attachments (C), the bronchiolar epithelial area (D), proportion of proliferating (E) and ciliated (F) cells in the bronchiolar epithelium, bronchiolar collagen content (G), and bronchiolar α-SMA content (H). Data points represent values from individual animals. Values with different letters are significantly different from each other (p<0.05). (PDF) [file pone.0159633.s002.pdf]

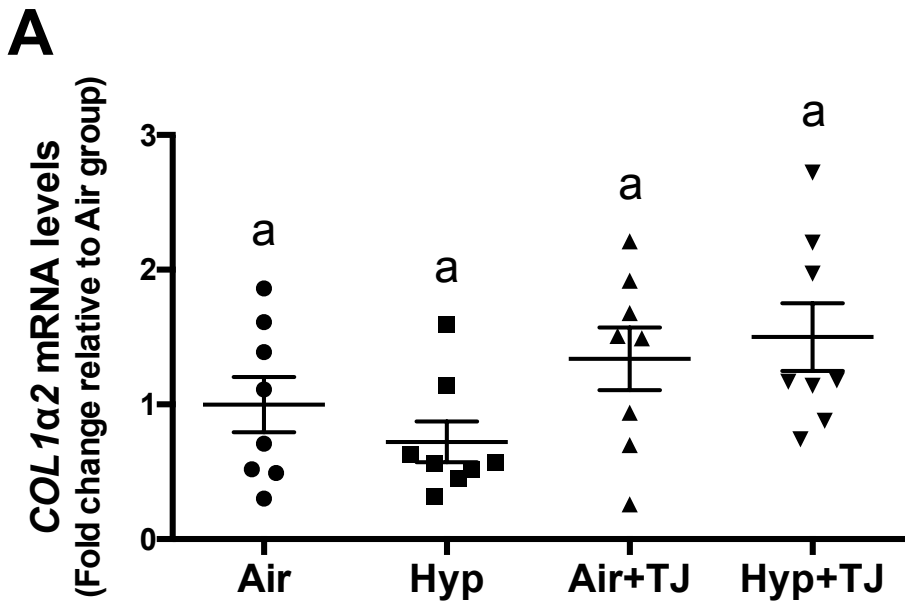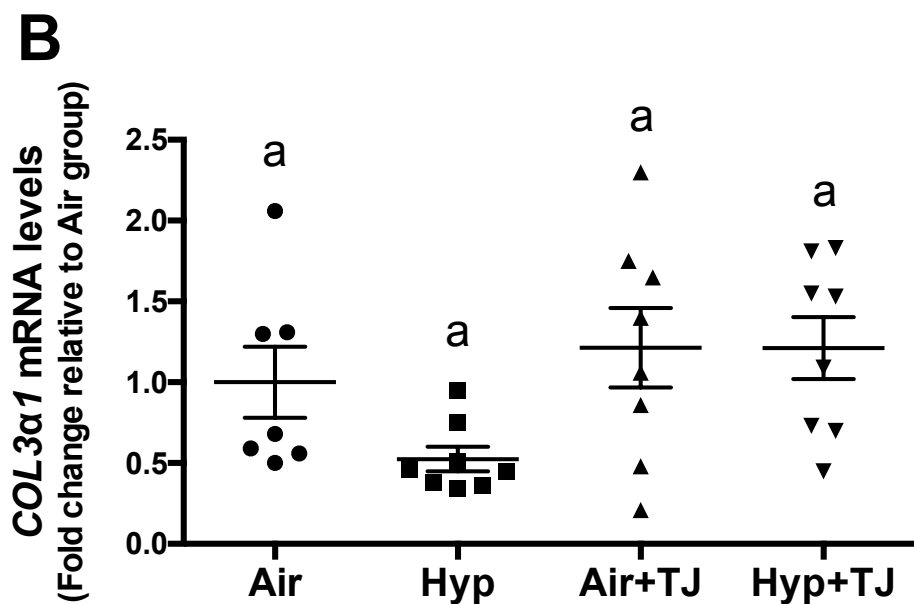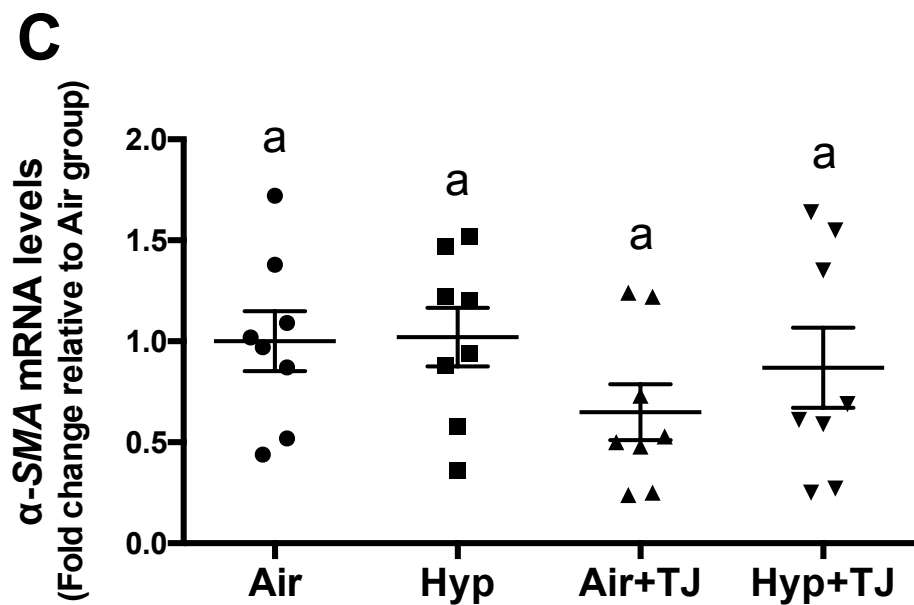

Supplement: S3 Fig — mRNA expression of COL1α2 (A), COL3α1 (B) and α-SMA (C) in lung tissue. Data points represent values from individual animals. Values with the same letter are not significantly different from each other (p<0.05). (PDF) [file pone.0159633.s003.pdf]

**A**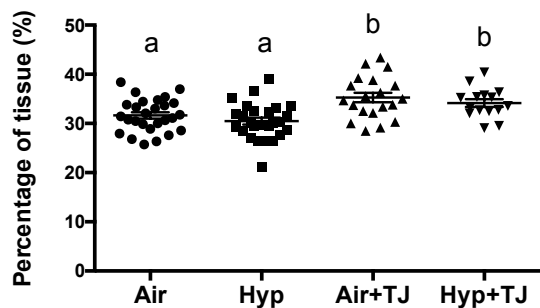**B**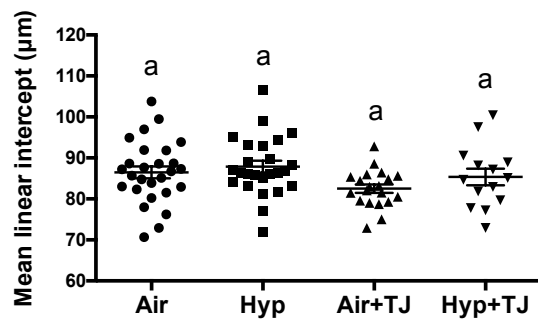**C**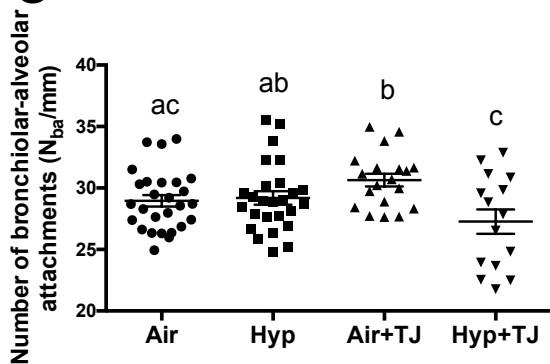**D**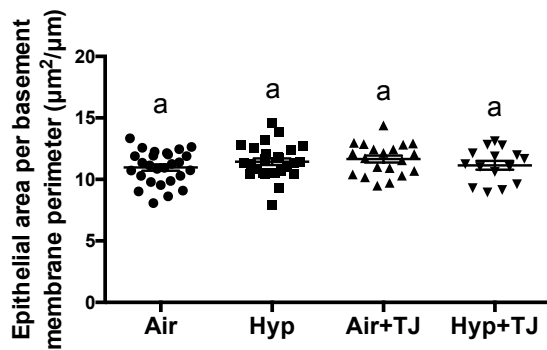**E**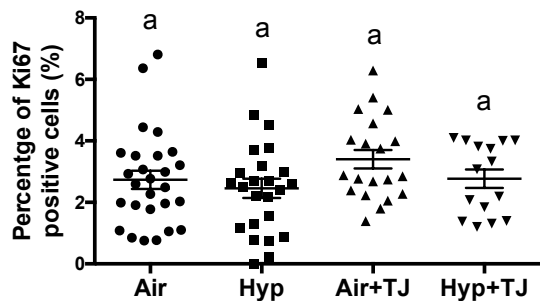**F**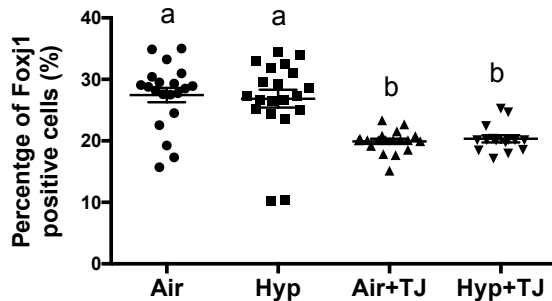

Supplement: S4 Fig — Morphometric analyses of tissue fraction (A), mean linear intercept (B), the number of bronchiolar-alveolar attachments (C), the bronchiolar epithelial area (D) and proportion of proliferating (E) and ciliated (F) cells in the bronchiolar epithelium. Data points represent values from individual animals. Values with different letters are significantly different from each other (p<0.05). (PDF) [file pone.0159633.s004.pdf]

**A**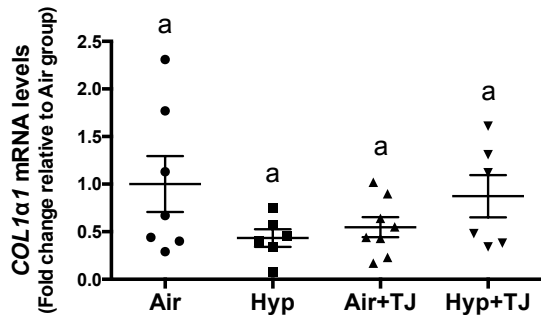**B**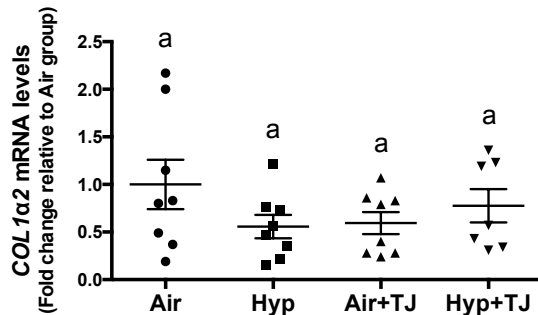**C**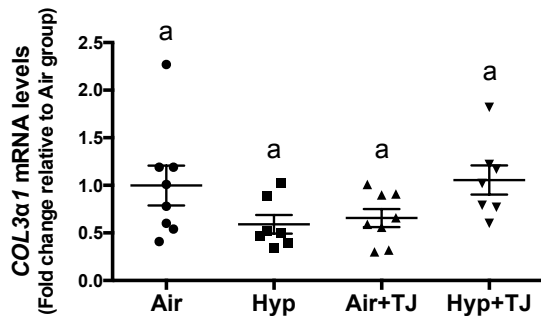**D**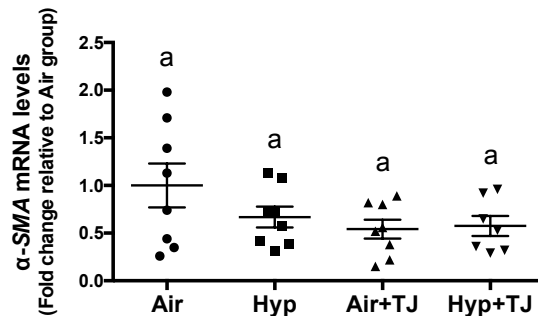

Supplement: S5 Fig — mRNA expression of COL1α1 (A), COL1α2 (B), COL3α1 (C) and α-SMA (D) in lung tissue. Data points represent values from individual animals. Values with the same letter are not significantly different from each other (p<0.05). (PDF) [file pone.0159633.s005.pdf]
